# *P. berghei* Telomerase Subunit TERT is Essential for Parasite Survival

**DOI:** 10.1371/journal.pone.0108930

**Published:** 2014-10-02

**Authors:** Agnieszka A. Religa, Jai Ramesar, Chris J. Janse, Artur Scherf, Andrew P. Waters

**Affiliations:** 1 Wellcome Trust Centre for Molecular Parasitology, Institute of Infection, Immunity and Inflammation, University of Glasgow, Glasgow, United Kingdom; 2 Leiden Malaria Research Group, Parasitology, Leiden University Medical Centre, Leiden, the Netherlands; 3 Biology of Host-Parasite Interactions Unit, Institut Pasteur, Paris, France; Université Pierre et Marie Curie, France

## Abstract

Telomeres define the ends of chromosomes protecting eukaryotic cells from chromosome instability and eventual cell death. The complex regulation of telomeres involves various proteins including telomerase, which is a specialized ribonucleoprotein responsible for telomere maintenance. Telomeres of chromosomes of malaria parasites are kept at a constant length during blood stage proliferation. The 7-bp telomere repeat sequence is universal across different *Plasmodium* species (GGGTTT/CA), though the average telomere length varies. The catalytic subunit of telomerase, telomerase reverse transcriptase (TERT), is present in all sequenced *Plasmodium* species and is approximately three times larger than other eukaryotic TERTs. The *Plasmodium* RNA component of TERT has recently been identified *in silico*. A strategy to delete the gene encoding TERT via double cross-over (DXO) homologous recombination was undertaken to study the telomerase function in *P. berghei*. Expression of both TERT and the RNA component (TR) in *P. berghei* blood stages was analysed by Western blotting and Northern analysis. Average telomere length was measured in several *Plasmodium* species using Telomere Restriction Fragment (TRF) analysis. TERT and TR were detected in blood stages and an average telomere length of ∼950 bp established. Deletion of the *tert* gene was performed using standard transfection methodologies and we show the presence of *tert* ^−^ mutants in the transfected parasite populations. Cloning of *tert-* mutants has been attempted multiple times without success. Thorough analysis of the transfected parasite populations and the parasite obtained from extensive parasite cloning from these populations provide evidence for a so called delayed death phenotype as observed in different organisms lacking TERT. The findings indicate that TERT is essential for *P. berghei* cell survival. The study extends our current knowledge on telomere biology in malaria parasites and validates further investigations to identify telomerase inhibitors to induce parasite cell death.

## Background

Telomerase is an RNA-dependent DNA polymerase complex functioning in extension and/or maintenance of telomeres [1] which are composed of a specialised conserved G-rich short (5-6 bp) tandem repeats [2], [3]. Telomeres are essential for maintenance of eukaryote chromosome integrity and stability since the telomeric DNA repeats and associated proteins prevent chromosome end-to-end fusions and exonuclease degradation (for a review see [4]). The length of telomeres is a critical determinant of a cell's replicative life span [5], [6] and telomere shortening has been linked to cell senescence, disease and ageing [5], [7]–[10]. Telomeres shorten with each cell division due to the so called end-replication problem [11], resulting in loss of telomeric repeats. The loss of telomeric repeats is compensated for by synthesis of new repeats. The enzyme responsible for synthesis of new repeats is the telomerase holoenzyme, which is an RNA-dependent DNA polymerase complex. This enzyme synthesises new tandem telomeric repeats *de novo* at the 3′ chromosome strand end [12]–[17]. Telomerase-negative cells (e.g. human somatic cells) experience telomere shortening and lose on average between 30 and 200 bp of telomeric sequence per cell division, a loss which is ultimately lethal [18]–[20].

Telomerase consists of several subunits. The core subunit consists of Telomerase Reverse Transcriptase (TERT) together with its conserved RNA component (Telomerase RNA; TR), which acts as the template for the synthesis of telomeric repeats [6]. Telomere structure is dependent on multiple telomere-associated proteins (TAPs) and these proteins together with telomeric DNA form the so-called telosome. TAPs are part of the telomerase-mediated telomere maintenance and regulation mechanisms, including telomere loop formation [21]. The gene encoding TERT has been deleted or mutated in a number of organisms which led to cell senescence and eventual cell death (“delayed death”) (e.g. [5], [6], [22]).

The genome of malaria parasites is arranged into 14 linear chromosomes which contain telomeres consisting of 7-bp telomeric repeat sequences (GGGTTT/CA) [23], [24]. The average telomere length per species varies, ranging from 850 bp (estimated ∼120 repeat copies) to 6700 bp (estimated ∼955 repeat copies) in the human parasites *P. falciparum* and *P. vivax*, respectively [25]. *Plasmodium* telomere length appears to be kept constant during the erythrocytic cycle [26] The genomes of different *Plasmodium* parasites contain a single copy *tert* gene. For TERT of *P. falciparum* (gene ID PF3D7_1314200) it has been demonstrated that it is capable of *de novo* synthesis of telomeric repeats both to the 3′ telomeric overhang and to non-telomeric 3′ ends, thus contributing not only to telomere maintenance but also to adding new telomeric sequences to broken chromosomes [27], [28]. Telomerase activity in *P. falciparum* is detectable both in late stage trophozoites and schizonts, stages where DNA replication occurs [27], [29]. The RNA component of telomerase (encoded by PF3D7_0918500 in *P. falciparum*; 2148 nt) has been identified *in silico* in several *Plasmodium* species based on structural comparisons of conserved domains in TR domains from other organisms [30]. The telomerase RNA is detectable in all erythrocytic stages and the ookinete stage of *P. falciparum* according to the PlasmoDB expression data (www.PlasmoDB.org).

In order to analyse the importance of telomere metabolism/dynamics for asexual blood stage multiplication of *Plasmodium* parasites we have attempted to generate mutants that lack expression of TERT. We have used the rodent malaria parasite *P. berghei* because of the high efficiency of transfection and rapid selection of gene-deletion mutants, which might be essential when TERT-deficient parasites show a delayed death phenotype as has been shown for other organisms. We found that we can target the *P. berghei tert* gene for gene deletion. However, our results also indicate that TERT is an essential enzyme for survival of *P. berghei* blood stages since we were unable to clone and propagate TERT-deficient parasites.

## Results

### Expression of PbTERT and PbTR in blood stages

**Figure 1:**
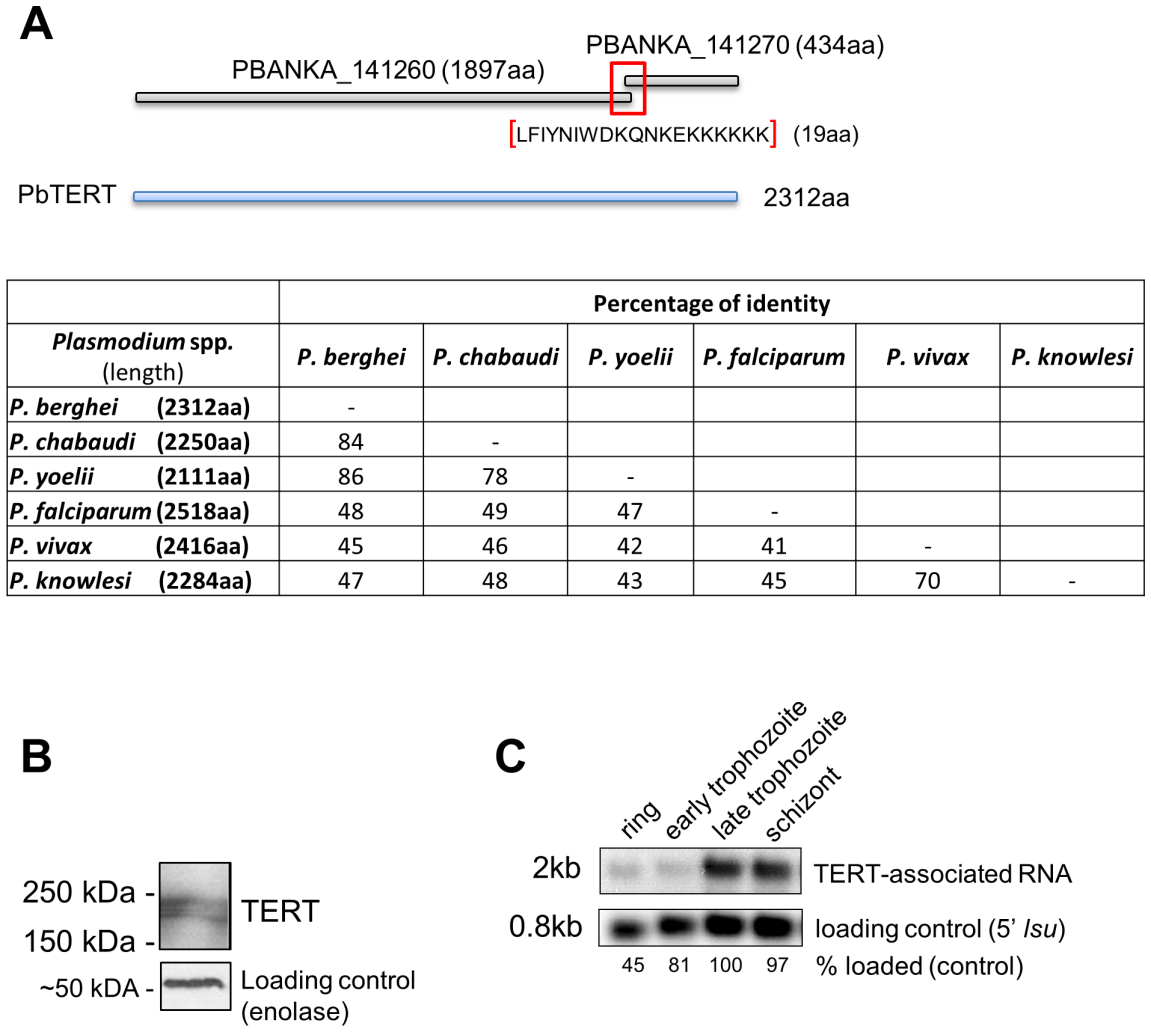
Pbtert gene structure (A) and PbTERT (B) and PbTR (C) expression. (A) The *tert* gene of *P. berghei* and homology (percentage identity) of TERT proteins in different *Plasmodium* species. Sequencing of the gap between two adjacent *tert* gene models available in PlasmoDB revealed a sequence duplication of 57 nt (19aa). The complete Pb*tert* gene encodes a protein of 2312aa, which is comparable to the size of other *Plasmodium tert* genes. (B) Western analysis of PbTERT protein in mixed blood stages. Two bands with a size between 150 and 250 kDa were detected (expected size of the TERT protein is ∼240 kDa). (C) Northern analysis of Telomerase-associated RNA (TR) in different blood stages of *P. berghei*. RNA was hybridized with a probe recognizing TR (upper panel) (the expected size of TR is 2 kb) and as a loading control with a probe recognizing *large subunit ribosomal RNA* (expected size 0.8 kb). The “% loading” refers to the quantity of the loading control signal detected for each stage relative to the “late trophozoite” lane which is set as 100%.

The *P. falciparum tert* gene sequence (PF3D7_1314200; 7557 bp) [29] was used to search the available *P. berghei* genome sequence. Two adjacent incomplete gene models have been identified in the genome of *P. berghei* (www.GeneDB.org) that encode *tert* genes: PBANKA_141260 (5691 bp) and PBANKA_141270 (1305 bp), that both share homology with the single copy *tert* genes of *P. falciparum* and *P. chabaudi* (PCHAS_141450; 6753 bp) (Fig. 1A). The *P. chabaudi tert* gene was subsequently used as a reference for assembling the complete *Pbtert* gene. Amplification of the predicted gap between the two *Pbtert* gene models using primers specific to the adjacent extremities of both gene models revealed a sequence duplication of 57 nt, which may explain the failure of correct assembly of *Pbtert* which has a total size of 6939 bp. The complete *Pbtert* gene shared 77%, 83% and 43% identity with the predicted *tert* genes of *P. yoelii* (17X), *P. chabaudi* and *P. falciparum*, respectively (table in Fig. 1A).

Expression of PbTERT in mixed blood stage parasites was assessed by Western blot analysis using anti-TERT antibodies raised against *P. falciparum* TERT [29] (Fig. 1B). In protein extracts of mixed blood stages two protein fragments hybridized to the antibodies; one with the expected size of PbTERT (240 kDa; 2312 aa) and a slightly smaller band of ∼220 kDa. The smaller band suggests that PbTERT is processed. Attempts to visualise PbTERT in blood stages by immunofluorescence microscopy using the same antibodies were unsuccessful.

The presence of TR transcripts (PBANKA_081945) was analysed by Northern analysis of RNA of different blood stages using a probe recognizing the complete transcript (Fig. 1C). Highest levels were detected in late trophozoites and schizonts consistent with the known expression pattern of TERT in *P. falciparum* blood stages [29] and the PlasmoDB expression data (www.PlasmoDB.org). TR was also readily detected in RNA collected from highly purified mature *P. berghei* gametocytes (data not shown). Our steady-state RNA sequencing data (Religa et al., in prep) shows a good correlation of TERT mRNA levels (rings, trophs, schizonts) with TR transcript levels obtained from Northern analysis (data not shown).

### Telomere length and localisation in blood stages

**Figure 2:**
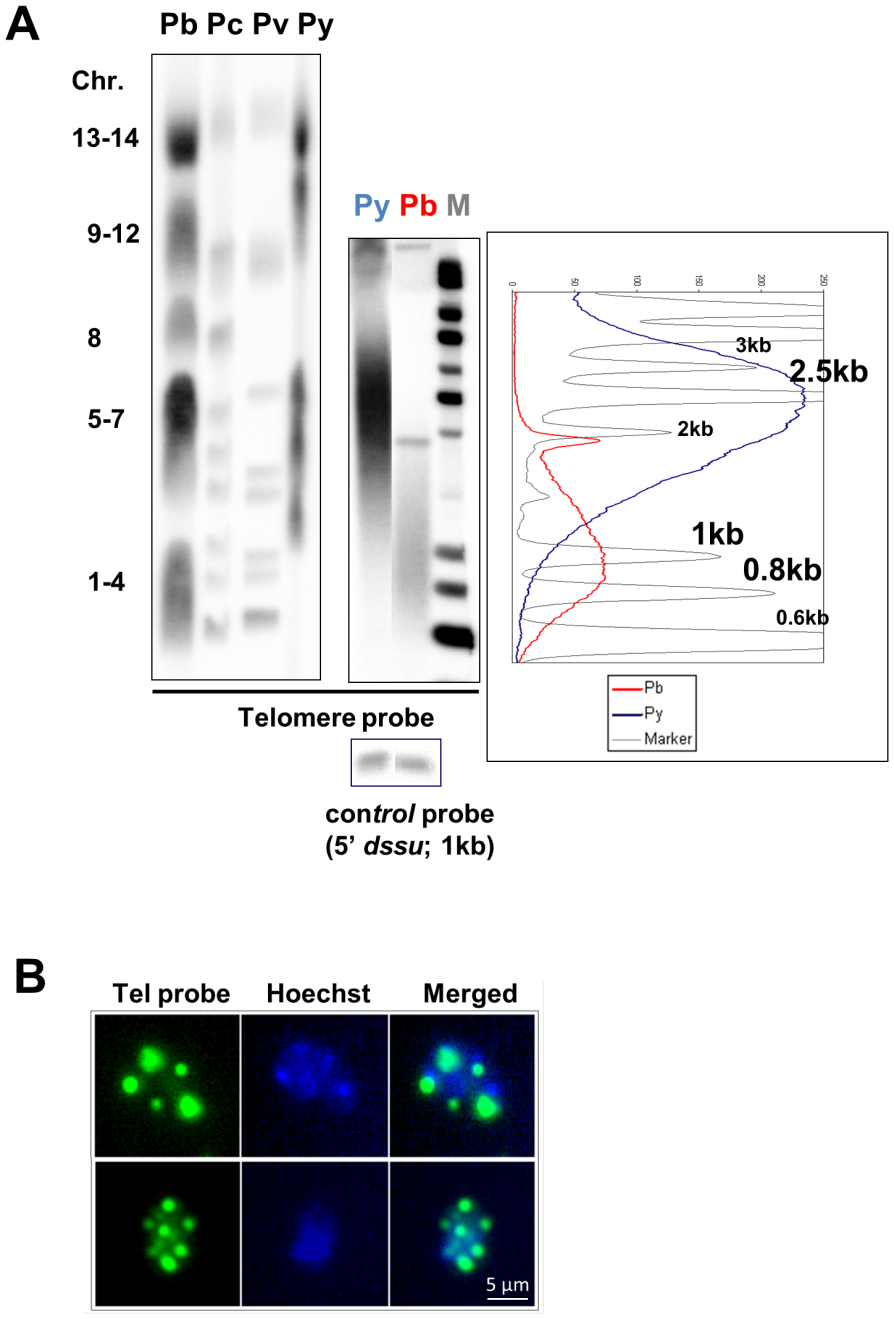
*P. berghei* telomere characterisation. (A) Determination of telomere length by Telomere Restriction Fragment (TRF) analysis. Left Panel: Southern analysis of separated chromosomes of *P. berghei* (Pb), *P. chabaudi* (Pc), *P. vinckei* (Pv) and *P. yoelii* (Py) showing hybridization of all chromosomes to a telomere-specific probe. The same probe was used for TRF analysis (middle, right panels). Middle panel: Southern analysis of digested *P. yoelii* (size control) and *P. berghei* gDNA probed with the telomeric probe showing the characteristic “smeared” hybridisation pattern in TRF analysis. Right panel: The average telomere length was measured as the highest peak of the signal intensity along the smear. Using the molecular marker (“M”, grey line) as a size reference (relevant marker bands sizes are noted on the graph), the mean telomere length was estimated to be ∼2500 bp and ∼950 bp for *P. yoelii* (blue line) and *P. berghei* (red line), respectively. Complete digestion of gDNA was confirmed by hybridisation with a 5′ *d-type small unit ribosomal RNA* probe. (B) Fluorescence *in situ* hybridisation with a telomere-specific probe. Fixed late blood stages of *P. berghei*. The telomeric probe (1.5 kb) was labelled with fluorescein (green). Hoechst (blue) was used for nuclear staining. The size bar is 5 µm.

The average telomere length of chromosomes in *P. berghei* and *P. yoelii* asexual blood stages was determined by telomere restriction fragment analysis (TRF) [31] using a previously described *Plasmodium* telomere-specific probe [32] that recognizes all chromosomes of the four rodent species, *P. berghei, P. chabaudi, P. yoelii* and *P. vinckei* (Fig. 2A, left panel). In *P. berghei* several chromosomes show a relatively strong hybridization signal (chromosomes 6, 7 and 13/14) as a result of additional ‘internal’ telomeric repeats in the subtelomeric 2.3 kb repeat element [33], [34]. The TRF analysis generated a characteristic ‘hybridisation smear’ in both *P. berghei* and *P. yoelii* (Fig. 2A, middle panel and the graph) which has also been observed in other *Plasmodium* species [35] and results from the variation in telomere length of chromosomes within a population of parasites. To determine the average telomere length, the signal intensity in each lane was plotted on the graph, with each lane coloured accordingly to the sample depicted (Fig. 2A). The predicted average telomere length was determined at around 950 bp for telomeres of *P. berghei*. This value is in agreement with previously determined telomere length of *P. berghei* chromosomes [36], [37] and is very similar to the telomere length of 960 bp reported for *P. chabaudi* [35]. The *P. yoelii* average telomere length is much higher and was determined to be approximately 2.5 kb, again similar to the size reported previously (approx. 2 kb) [35]. Fluorescence *in situ* hybridization (FISH) using a fluorescein-labelled telomere sequence-specific probe [35] revealed a clear nuclear foci signal in the nuclei of late blood stages of *P. berghei* (Fig. 2B). However, the analysis of trophozoite nuclei also suggests the presence of clusters of telomeres within a single nucleus as has been shown for blood stages of *P. falciparum* (Fig. 2B) [38].

### Deletion of the tert gene and selection of tert-deletion mutants

**Figure 3:**
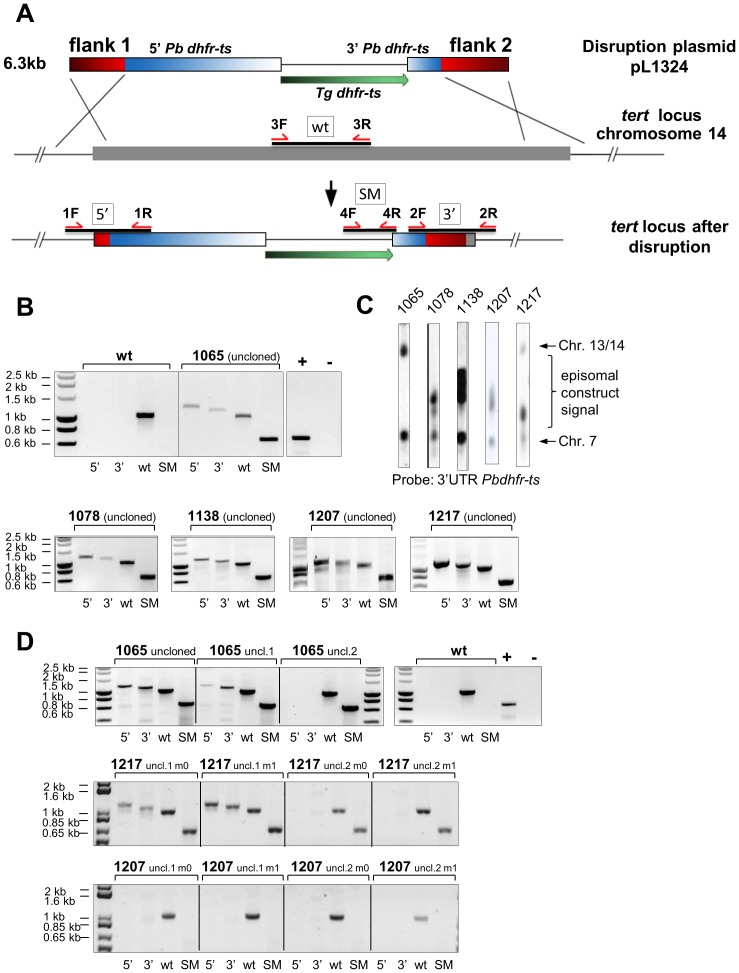
Pbtert deletion and selection of tert- mutants. (A) Schematic representation of the construct used to delete the *tert* gene. The construct, containing the *Tgdhfr-ts* selectable marker (SM) cassette, targets the *tert* gene at the flanking regions (red) by double cross-over integration. The red arrows indicate primers used for diagnostic PCR to confirm correct disruption of *tert*. Boxes correspond to lanes on the PCR gels in (B), (D) and Fig. S1A. (B) Diagnostic PCR of uncloned parasites transfected with a DNA construct to delete the *tert* gene. Parasites were collected and analysed directly after transfection and selection with pyrimethamine (parent populations). Diagnostic PCR shows the presence of parasites with correct disruption of the *tert* gene. In all experiments (1065, 1078, 1138, 1207, 1217) the 5′ and 3′ integration fragments (lanes 5′, 3′), as well as the *Tgdhfr-ts* fragment (lane SM) were amplified. However, all populations contained parasites with a wild type *tert* gene as shown by amplification of the wild type *tert* fragment (lane wt). The primer pairs used are shown in (A) and expected fragment sizes in Table S2. *pbs21*-specific primers were used as a positive control for all the PCR reactions (“+”). The water control is marked as “-“. (C) Southern analysis of separated chromosomes using the 3′UTR *Pbdhfr-ts* probe shows only in experiment 1065 and 1217 hybridisation with chromosome 14 on which the *tert* gene is located. This probe recognizes the endogenous *Pbdhfr-ts* gene on chromosome 7 in all populations and additional chromosomes in experiments 1078, 1138, 1207 (possible episomal construct signal). (D) Diagnostic PCR of uncloned and propagated parasites transfected with a DNA construct to delete the *tert* gene. The parent parasite populations of experiment 1065, 1207 and 1217 [see (B)] were propagated in mice (m0  =  mouse 0, m1  =  mouse 1) for another 1–2 weeks. Parasite populations collected were analysed by diagnostic PCR for the presence of parasites with correct disruption of the *tert* gene [primers same as in (B)]. In all populations no parasites with a disrupted *tert* gene could be detected by diagnostic PCR after 1 week (1207 all populations) or after two weeks of propagation (1065 uncl.2, 1217 uncl.2 m0 and 1217 uncl.2 m1).

In order to delete the *tert* gene of *P. berghei* we designed a standard double cross-over plasmid (pL1324) based on plasmid pL0001 plasmid (www.mr4.org: plasmid MRA-770, www.pberghei.eu). Plasmid pL1324 contains the *Tgdhfr-ts* selection cassette flanked by two genomic sequences of ∼750 and 950 bp of the *tert* gene. The schematic representation of the construct and the expected integration event that leads to deletion of *tert* is shown in Fig. 3A. Wild type (WT) parasites were transfected using standard methods of transfection [39]. In five independent transfection experiments (exp. 1065, 1078, 1138, 1207, 1217), parasites were selected with pyrimethamine and parasite populations were collected between day 10–14 after infection with transfected parasites (at a parasitaemia of 2–5%). These parasite populations were genotyped by Southern analysis of separated chromosomes and diagnostic PCR analysis. Diagnostic PCR analysis clearly showed the presence of a correctly disrupted *tert* gene in all five parasite populations (Fig. 3B). However, these populations also consisted of parasites with a WT *tert* gene as shown for all experiments (Fig. 3B, lane wt). Southern analysis of separated chromosomes using a construct specific probe (*3*′*-UTR Pbdhfr-ts)* revealed in 3 experiments no or very weak hybridisation signals at chromosome 14 where the *tert* locus is located (Fig. 3C), indicating that the ratio of *tert*-deletion parasites and parasites with a WT-copy of *tert* is very low. In three experiments (1078, 1138, 1207) the intensity of the hybridization signal (compared to the signal of chromosome 7 on which the endogenous *Pbdhfr-ts* gene is located) and ‘smeary’ appearance of the signal is indicative of the presence of episomes containing the selectable marker (SM) cassette. Such episome-containing parasites are mainly selected when genes are targeted that are either essential for blood stage growth or strongly affect growth, resulting in selecting those rare parasites containing multiple copies of the episomes. However, a clear hybridisation with chromosome 14 was observed in experiment 1065 and 1217 (Fig. 3C). We therefore undertook large cloning experiments (20 mice per cloning experiment) to collect pure populations of *tert*-deletion mutants. However the attempts to clone *tert*-deficient parasites were unsuccessful and the only clones derived were those that contained an intact *tert* gene as shown by diagnostic PCR analysis (1065 clones 1–9, Fig. S1A). Interestingly, Southern analysis of chromosomes of the clones of 1065 line still exhibited evidence for integration of the construct in chromosome 14 suggesting an incorrect integration event of the construct occurred that introduced the selectable marker *Tgdhfr-ts* on chromosome 14 but left the *tert*-gene intact (Fig. S1B). Integration of the *Tgdhfr-ts* gene in chromosome 7 has been excluded by both Southern and PCR analysis of the experiment 1065 clones (Fig. S1B and C, respectively). Such integration events have been observed before in selected parasite populations after transfection with constructs that target genes that result in lethal or affected-growth phenotypes (unpublished observations CJJ). Possible retention of the *tert* deletion plasmid was excluded (Fig. S2). The failure to clone the *tert*-deletion mutants might be explained by a low percentage of *tert*-deletion parasites in the populations, thereby reducing the chance of infecting a mouse with a single mutant. However, it is also possible that some mice have been injected with a single *tert*-deletion parasite but these mice became negative after the growth period of 8 days because the mutant parasites died as a result of telomere shortening during each multiplication cycle.

In order to check if *tert*-mutants were disappearing from populations that undergo asexual multiplication, we injected 10^5^ parasites of the uncloned populations of 1065, 1217 and 1207 in two mice and analysed the presence of *tert*-deletion parasites by diagnostic PCR during a period of 1–2 weeks of asexual multiplication. In all three populations the *tert*-deletion parasites could not be detected at the end of 1–2 week period (Fig. 3D). These results indicate that *tert*-deletion mutants have a growth disadvantage compared to parasites with a WT *tert* copy, possibly due to a delayed cell death phenotype of *tert*-deletion mutants.

## Discussion

This study reports an analysis of telomeres and telomerase of the rodent malaria parasite *P. berghei* and the unsuccessful attempts to isolate gene-deletion mutants lacking the gene encoding telomerase reverse transcriptase (TERT). Although we are able to demonstrate the presence of *tert*-deficient parasites in transfected populations we were unable to clone these parasites indicating that TERT is essential for *P. berghei* cell survival in keeping with all studies reported to date on the essential role of TERT for survival of dividing cells of other eukaryotes (e.g. [40]–[42] and references therein).

Our inability to clone *tert*-deficient parasites suggests that the absence of TERT strongly affects the viability of blood stages and the rapid disappearance of *tert*-deficient parasites from dividing cell populations suggests a ‘delayed cell-death’ phenotype. The parasite lines obtained following parasite cloning all exhibited a possible non-specific integration event of the *tert* KO transfection construct (Fig. S1B, C), whereas *tert* KO plasmid presence was excluded (Fig. S2). Based on human cell studies where in the absence of TERT shortening of telomeres occurs at a rate of 50–100 bp per cell division [43], [44] and assuming lethality in *tert*-deficient parasites occurs only after the complete loss of telomeres, the approximate survival time of the *P. berghei tert*-deficient parasites can be calculated. The number of nuclear/cell divisions in *P. berghei* is 3–4 per 24 hours. With a (mean) length of 950 bp and a loss of 50–100 bp per nuclear division, telomeres will be lost after 10-19 nuclear divisions which equals 2.5–6 days of multiplication in mice. This rate of telomere loss might explain the failure to obtain cloned parasite lines which takes a period of 14–18 days after transfection. However there exists the possibility that telomere shortening in *Plasmodium* has different kinetics than in other dividing cells. Generation of conditional KO parasites where *tert* can be synchronously disrupted in a homogenous population of transfected parasites might help to better define telomere turn-over in *Plasmodium*. The mean telomere length is not the same in different *Plasmodium* species and can differ significantly. For example *P. vivax* telomeres have an estimated length of 6.7 kb and *P. yoelii* 2–2.5 kb, which is significantly longer than the 950 bp of *P. berghei* telomeres (this paper; [35]). If telomere shortening per nuclear division is similar in *P. berghei* and *P. yoelii* it might therefore be possible to successfully clone *P. yoelli tert*-deficient parasites. However, TERT is possibly not only involved in telomere synthesis. TERT has been shown to interact with a range of other proteins in other organisms [45] and hence the absence of TERT might affect other pathways that are not necessarily associated with telomere shortening and chromosome instability. The function of TERT in telomere synthesis is dependent on the conserved RNA component of the TERT complex (TR). *Plasmodium* TR has first been identified in *P. falciparum*. The *P. berghei* orthologous molecule (PBANKA_081945) has been annotated based on strand specific transcriptome data and synteny to *P. falciparum* (U. Boehme, pers. comm.; www.GeneDB.org). Abolishing TR might be an alternative for the inactivation of the function of the TERT complex and might have a less strong lethal effect on blood stages compared with deletion of TERT. However, in other organisms cell dysfunctions resulting from the absence of TERT and TR loss overlap (e.g. [46]). Other approaches, such as the generation of parasite lines that carry mutated forms of TERT/TR or conditional mutagenesis [5], [6], [47]–[50] to inactivate TERT, might provide more insight into the role of TERT in telomere addition and help to define the minimal length of *Plasmodium* telomeres for chromosome stability, cell survival and delayed cell death. The availability of parasites that have a delayed cell death phenotype and that would result in self-resolving infections might also be useful tools for studies on the use of attenuated blood stage parasites to induce protective immune responses [51]. Such parasites would allow for analysing immune responses after (repeated) exposure to low numbers of replicating parasites without the need for clearing infections with antimalarials [52].

The studies reported here reveal TERT to be an essential protein for maintenance of *Plasmodium* blood stage parasite viability and can therefore be a legitimate target for the development of antimalarial drugs. Non-nucleoside reverse transcriptase analogues (NNRTIs) have been shown to kill *P. falciparum in vitro* after 3–5 blood stage cycles (A. Scherf, unpublished data), and efficiently inhibit *P. yoelii* liver stages *in vitro* [53] showing/indicating that *Plasmodium* TERT complex/activity can be targeted.

## Materials and Methods

### Experimental animals and parasite reference lines

All animal experiments performed in the Leiden malaria Research Group were approved by the Animal Experiments Committee of the Leiden University Medical Center (DEC 10099). All surgery was performed under isofluorane anaesthesia, and all efforts were made to minimise suffering. For all experiments the reference line cl15cy1 of the ANKA isolate of *P. berghei* was used [39].

### Completion of the Pbtert gene sequence

The Pbtert sequence gap was amplified with primers GU2046 and GU2045 (see Table S1 for sequences), specific to the available *P. berghei* partial genes PBANKA_141260 and PBANKA_141270, respectively. The obtained PCR product was subcloned into pCR 2.1 TOPO vector (Invitrogen) and the inserts sequenced. Sequence assembly was performed in CLC Genomics Workbench (CLCbio).

### Pbtert KO vector generation

The two homology arms of the TERT double cross-over (DXO) plasmid were amplified from *P. berghei* gDNA by PCR using primer pairs 3298/3299 [homology arm(HA) 1] and 3300/3301 [homology arm (HA) 2] (Table S1 primer sequences). PCR products were digested with Asp718I/HindIII (HA 1) and EcoRI/XbaI (HA 2) and subsequently cloned into the standard pL001 plasmid which contains the Toxoplasma gondii dihydrofolate reductase – thymidylate synthase gene (Tgdhfr-ts) conferring resistance to pyrimethamine [54]. The final TERT knockout vector (pL1324) was linearised with Asp718I and XbaI restriction enzymes, the 6.3 kb tert KO DNA fragment gel-purified and approximately 5 µg were used for transfection in order to obtain double cross-over tert knockout parasites.

### *P. berghei* transfection, construct integration validation

Transfection of *P. berghei* parasites was performed as described [39]. Briefly, purified schizonts were transfected by electroporation and injected i.v. into a mouse tail (day 0). Pyrimethamine drug treatment was implemented at day 1. Parasites were collected at day 10–15 (parasitaemia of 2–5%) by cardiac puncture under anaesthesia. Erythrocytes were lysed in a cold 1× erythrocyte lysis buffer (10× stock: 1.5M of NH_4_Cl, 0.1M KHCO_3_, 0.1M EDTA in H_2_O). Genomic DNA was extracted from blood stage parasites and analysed for construct integration by diagnostic PCR using the following primer pairs 1F/1R and 2F/2R (5′ and 3′ integration, respectively), 3F/3R (Pbtert gene fragment), 4F/4R (Tgdhfr-ts) (see Table S2 for expected products and Table S1 for primer sequences).

### Telomere Restriction Fragment (TRF) analysis

Telomere Restriction Fragment analysis was performed as previously described [35] with modifications. 700 ng of gDNA was digested overnight at 37°C with AluI, MboII, RsaI and Sau3AI (5 units each), run on 1% agarose gel and blotted onto a positively charged nitrocellulose membrane (Hybond-N^+^ membrane, GE Healthcare). The membrane was washed shortly in 2× SSC, pre-hybridised for at least 1 hour at 65°C, and probed with α-^32^P dATP labelled double stranded telomere-specific probe from pTB4.1 plasmid [32] for 6 hours at 60°C. The blot was washed 3 times with 3× SSC/0.5% (v/v) SDS and once with 1× SSC/0.5% (v/v) SDS at 60°C (15 min. each wash), exposed to a Biorad phosphoimaging screen-K and results scanned using Typhoon 9410 from GE Healthcare. Image analysis was performed using ImageQuant TL (Amersham Biosciences).

### Northern blot analysis

Northern analysis of total RNA obtained from synchronised *P. berghei* blood stages was performed as described previously [55], [56]. Approximately 5 µg of RNA was hybridized to the P^32^ labelled DNA probe for telomerase-associated-RNA that was PCR amplified from wild type *P. berghei* gDNA using the primer set 3306/3307 (see Table S1). As a loading control primer L644R was used, which hybridizes to the blood stage large subunit ribosomal RNA [57]. The blots were exposed to a Biorad phosphoimaging screen-K and scanned using Typhoon 9410 (GE Healthcare). Quantification of the loading control signal intensities was done using ImageJ.

### Pulse-field gel (PFG) electrophoresis

Separation of chromosomes by PFG electrophoresis was performed as previously described [39]. The blots were exposed to a Biorad phosphoimaging screen-K and scanned using Typhoon 9410 (GE Healthcare).

### Western analysis

For Western analysis mixed *P. berghei* blood stages (2×10^7^ parasites) were resuspended in 2× SDS gel-loading buffer (100 mM Tris-HCl pH 6.8; 200 mM dithiothreitol; 4% SDS; 0.2% bromophenol blue; 20% glycerol) with prior addition of 1 µl of PMSF (C_i_ = 100 mM, C_f_ = 1 mM). The samples were run on a 12% SDS-PAGE gel and transferred to a nitrocellulose membrane, which was subsequently blocked with 5% milk/0.1% Tween in PBS, probed with the PfTERT primary antibody [29] in 5% milk/0.1% Tween in PBS (rabbit, 1∶500, courtesy of A. Scherf), washed 3× with 0.1% Tween/PBS and probed with an HRP-labelled secondary goat anti-rabbit antibody (Dako) in 5% milk/0.1% Tween in PBS (1∶5000). Same procedure was used for the control antibody against enolase (PBANKA_121430; rabbit, 1∶1000, Biogenes). The complex was visualised using the ECL Plus™ Western blotting detection reagents (Amersham Biosciences), and X-ray film (Kodak).

### Fluorescent in situ hybridization (FISH)^DNA-DNA^

FISH analysis used for telomeric DNA detection in *P. berghei* blood stage parasites was performed as described [32], [38], omitting the step of saponin lysis. Fluorescence emission was analysed using a Leica DMRA Fluorescence microscope and ColourProc software.

## Supporting Information

Figure S1 **(A) Diagnostic PCR of 1065 clones 1–9.** The 5′ and the 3′ integration fragments (lanes 5′ and 3′) were not amplified. The wild type *tert* (lane wt) and *Tgdhfr-ts* (lane SM) fragments were obtained for all clones and the uncloned population. Primers used and amplified products are same as in Fig. 3B and D. Schematic representation of the modified *tert* locus indicating the primers and products is shown in Fig. 3A. For primers and expected sizes see Table S2. **(B) Southern analysis of separated chromosomes in experiment 1065 clones 1, 2 and 4–9** using the 3′UTR *Pbdhfr-ts* probe shows hybridisation with chromosome 7 and 14. The *Tgdhfr-ts* probe shows the signal only in chromosome 14. **(C) Diagnostic PCR for** ***Tgdhfr-ts*** **presence in chromosome 7 in experiment 1065 clones 1–3**. 5′ and 3′ integration fragments (lanes 1, 2) were not amplified. Unspecific PCR product (lane 1) is caused by the GC-rich L301 primer as shown in PCR with both primers, and with each primer separately (lane 1, 3580 primer – lane 1a, L301 primer – lane 1b). The wt *tert* and *Tgdhfr-ts* fragments (lanes wt, SM) were amplified. For primers and expected sizes see Table S2. The “+” and “-“ control primers same as in Fig. 3B.
(PDF)

Figure S2 **PCR for** ***tert*** **KO plasmid presence in experiment 1065 clones 1–3.** *tert* KO plasmid control fragments are not amplified in either of 1065 samples (lanes A, B). The *tert* and *Tgdhfr-ts* gene fragments (lanes wt, SM) are amplified in all 1065 samples and not in the *tert* KO plasmid. Schematic representation of the *tert* construct is shown indicating primers used (see Table S2 for expected products sizes).
(PDF)

Table S1 **A list of primers used in this study.**
(PDF)

Table S2 **Primer combinations and expected product sizes in PCR analyses performed in this study.**
(PDF)
