# Supplementary figures and images for: P. berghei Telomerase Subunit TERT is Essential for Parasite Survival
Source: PLoS One. 2014 Oct 2;9(10):e108930. doi: 10.1371/journal.pone.0108930 (PMC4183507; doi:10.1371/journal.pone.0108930)

Figure S1

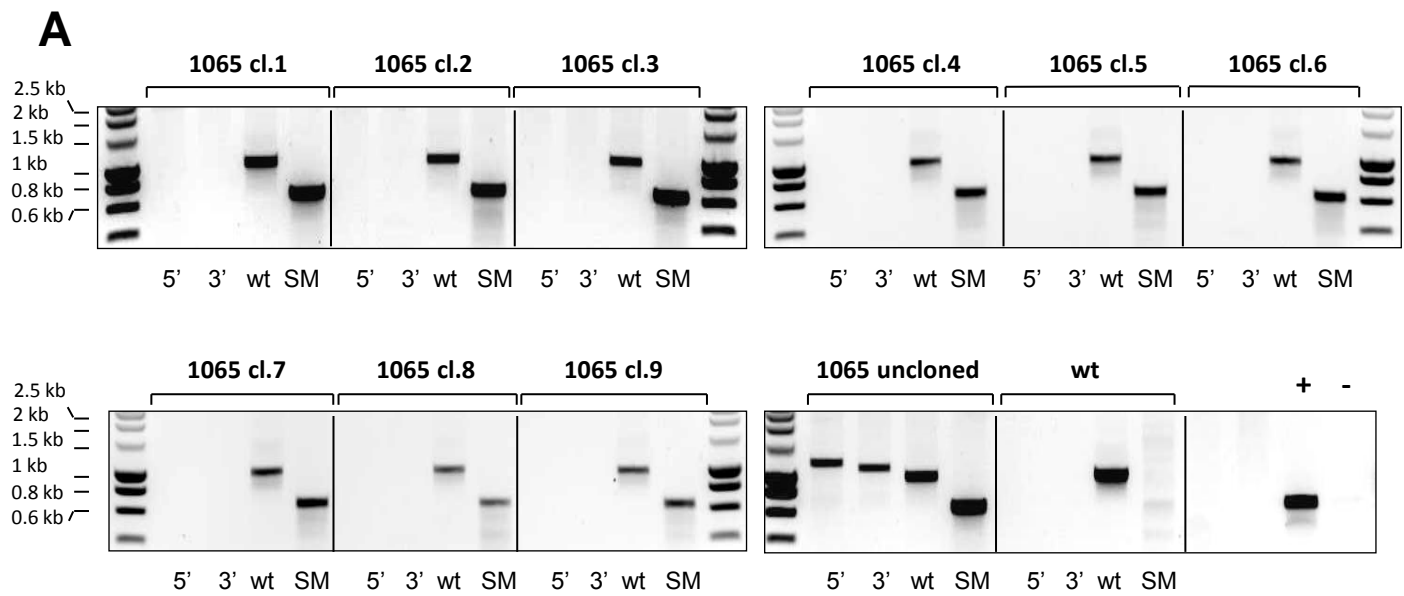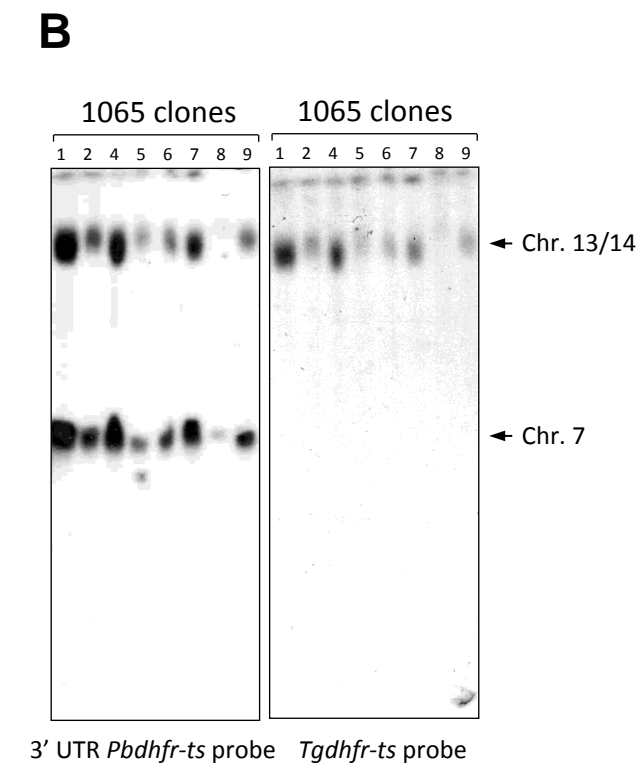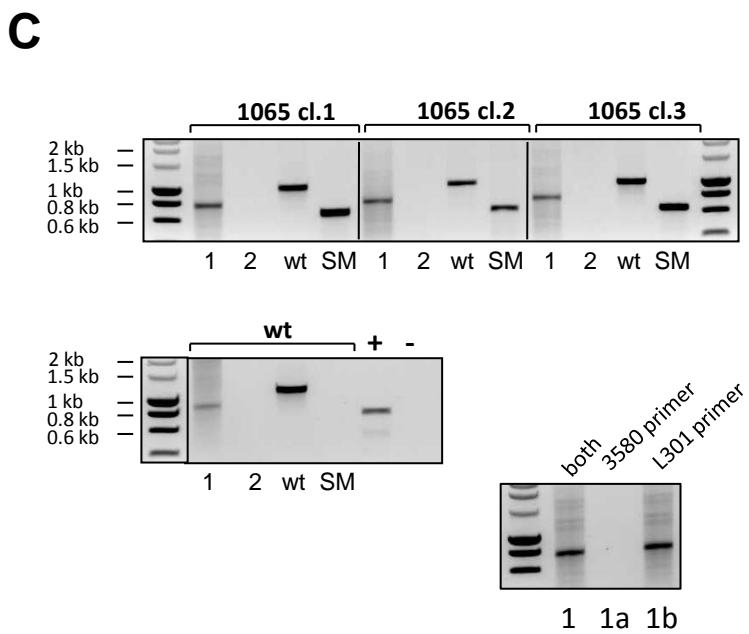

Supplement: Figure S1 — (A) Diagnostic PCR of 1065 clones 1–9. The 5′ and the 3′ integration fragments (lanes 5′ and 3′) were not amplified. The wild type tert (lane wt) and Tgdhfr-ts (lane SM) fragments were obtained for all clones and the uncloned population. Primers used and amplified products are same as in Fig. 3B and D. Schematic representation of the modified tert locus indicating the primers and products is shown in Fig. 3A. For primers and expected sizes see Table S2. (B) Southern analysis of separated chromosomes in experiment 1065 clones 1, 2 and 4–9 using the 3′UTR Pbdhfr-ts probe shows hybridisation with chromosome 7 and 14. The Tgdhfr-ts probe shows the signal only in chromosome 14. (C) Diagnostic PCR for Tgdhfr-ts presence in chromosome 7 in experiment 1065 clones 1–3. 5′ and 3′ integration fragments (lanes 1, 2) were not amplified. Unspecific PCR product (lane 1) is caused by the GC-rich L301 primer as shown in PCR with both primers, and with each primer separately (lane 1, 3580 primer – lane 1a, L301 primer – lane 1b). The wt tert and Tgdhfr-ts fragments (lanes wt, SM) were amplified. For primers and expected sizes see Table S2. The “+” and “-“ control primers same as in Fig. 3B. (PDF) [file pone.0108930.s001.pdf]

Figure S2

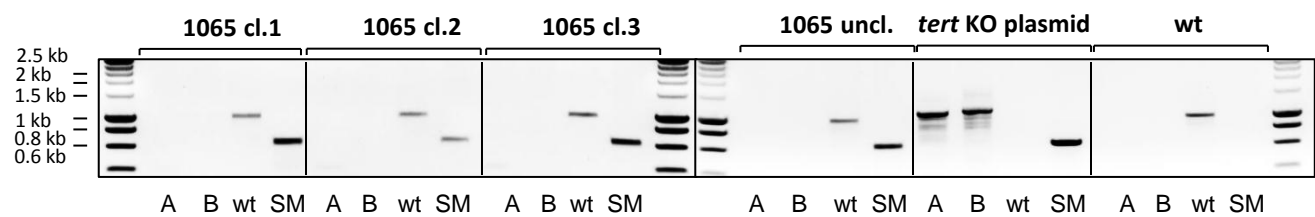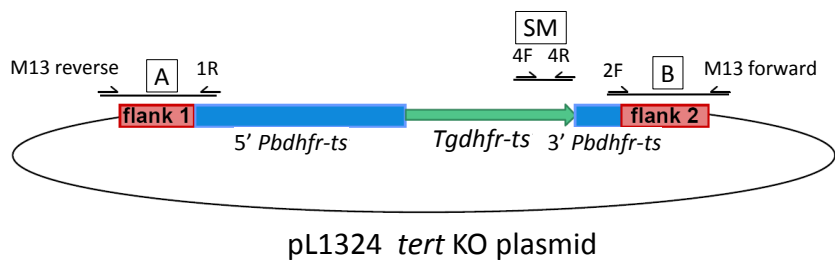

Supplement: Figure S2 — PCR for tert KO plasmid presence in experiment 1065 clones 1–3. tert KO plasmid control fragments are not amplified in either of 1065 samples (lanes A, B). The tert and Tgdhfr-ts gene fragments (lanes wt, SM) are amplified in all 1065 samples and not in the tert KO plasmid. Schematic representation of the tert construct is shown indicating primers used (see Table S2 for expected products sizes). (PDF) [file pone.0108930.s002.pdf]
