# Supplementary material for: P. berghei Telomerase Subunit TERT is Essential for Parasite Survival
Source: PLoS One. 2014 Oct 2;9(10):e108930. doi: 10.1371/journal.pone.0108930 (PMC4183507; doi:10.1371/journal.pone.0108930)
Supplement: Table S1 — A list of primers used in this study. (PDF) [file pone.0108930.s003.pdf]

Table S1

Primer	Sequence (5' to 3')
1F	GTCAGAACATAGAATTAGTTATG
1R	AATATTCATAACACACTTTTAAGC
2F	GATTCATAAATAGTTGGACTTG
2R	CATGGAATAATTAACAAACTCG
3F	AGGTGATAAAATAGAAAC
3R	TGAATTATTTAGCCATTC
4F	AGATCTGGGACAAGAATGTG
4R	ACTAGTGACAGCCATCTCCATCTG
GU2045	GAAAGATATTTCGAGACAATCTTTTC
GU2046	CCTCTTATTTATAAAAGTGATTTATTTATTTAC
L301	ATCGCGACGACCAGACACAC
3306	CGCTATTTTATCTCTCAAG
3307	CCAACCATCTATAAGGAG
3298	AAAggtaccATTTCTTTTATCCTACAC
3299	AAAaagcttTGTTTTTTAAGTAACTAC
3300	AAAgaattcCTATCAGATCTATTGAAT
3301	AAAtctagaTAATAATTTGCTGAACAG
3580	GTATACAATATATATCATGGG
3581	GTGTAGATAAAGAACACCTTG
