# Supplementary material for: P. berghei Telomerase Subunit TERT is Essential for Parasite Survival
Source: PLoS One. 2014 Oct 2;9(10):e108930. doi: 10.1371/journal.pone.0108930 (PMC4183507; doi:10.1371/journal.pone.0108930)
Supplement: Table S2 — Primer combinations and expected product sizes in PCR analyses performed in this study. (PDF) [file pone.0108930.s004.pdf]

Table S2

***tert* KO analysis**			Expected	
Lane	Primers used	Product (kb)	wt	*Pbtert* KO
5’	1F/1R (5' integration)	1.2	-	+
3’	2F/2R (3' integration)	1.1	-	+
wt	3F/3R (*Pbtert*)	1	+	-
SM	4F/4R (*Tgdhfr-ts*)	0.6	-	+

**Fig. S1C *Tgdhfr-ts* presence in chr.7 analysis**			Expected	
Lane	Primers used	Product (kb)	wt	*Pbtert* KO
1	3580/L301 (5' integration)	3	-	+
2	1F/3581 (3' integration)	1.1	-	+
wt	3F/3R (*Pbtert*)	1	+	-
SM	4F/4R (*Tgdhfr-ts*)	0.6	-	+

**Fig. S2 *tert* KO plasmid presence analysis**			Expected		
Lane	Primers used	Product (kb)	wt	*Pbtert* KO	*tert* KO plasmid
A	M13 rev/1R (5' cassette)	1.1	-	-	+
B	M13 for/2F (3' cassette)	1.16	-	-	+
wt	3F/3R (*Pbtert*)	1	+	-	-
SM	4F/4R (*Tgdhfr-ts*)	0.6	-	+	+
